# Real-world outcomes of combined therapy of photodynamic therapy with anti-vascular endothelial growth factor for polypoidal choroidal vasculopathy

**DOI:** 10.1038/s41433-021-01773-x

**Published:** 2021-09-28

**Authors:** Siyin Liu, Ramandeep Chhabra

**Affiliations:** 1 Manchester Royal Eye Hospital, Manchester University NHS Foundation Trust, Manchester, UK; 2 School of Biological Sciences, Faculty of Biology, Medicine and Health, University of Manchester, Manchester, UK; 3 Manchester Academic Health Science Centre, Manchester, UK

**Keywords:** Macular degeneration, Drug therapy

## Abstract

**Objectives:**

To describe the real-world outcomes of photodynamic therapy (PDT) as a rescue therapy in eyes with polypoidal choroidal vasculopathy (PCV) refractory to anti-vascular endothelial growth factor (VEGF) monotherapy in a British cohort of patients.

**Methods:**

This is a retrospective chart review of 53 eyes with PCV. Based on the timing of PDT, the eyes were stratified into two groups (9 in the Initial-PDT group, 44 in the Deferred group). The number of anti-VEGF injections/year and the best corrected visual acuity (BCVA) before and after PDT were analysed. Multivariate regression model was created to identify factors predictive of visual outcome and treatment burden after PDT.

**Results:**

The Deferred group received a mean of 9.4 injections/year but significantly reduced to 7.2 after PDT (*p* < 0.001). The Initial-PDT group required significantly fewer injections after PDT compared to the Deferred group (*p* = 0.004). The Deferred group experienced improvement in BCVA from 58.7 letters at baseline to 63.8 at 18-months follow-up (*p* < 0.001), but no significant increase was observed in the Initial-PDT group (*p* = 0.310). Better baseline BCVA is associated with higher likelihood of achieving good BCVA ≥ 70 letters after PDT (Odd Ratio=1.12, 95% CI: 1.03–1.21, *p* = 0.006), while increased number of anti-VEGF injections/year before PDT reduces the likelihood of easing treatment burden to ≥12 weeks apart between each injection after PDT (Odd Ratio=0.724, 95% CI: 0.58–0.91, *p* = 0.006).

**Conclusions:**

PDT as a rescue therapy is beneficial in the long-term management of PCV, particularly in eyes that had experienced a significant period of prior exposure to anti-VEGF monotherapy.

## Introduction

Polypoidal choroidal vasculopathy (PCV) is an exudative maculopathy characterised by the presence of terminal aneurysmal polypoidal lesion with or without anomalous branching network of choroidal vessels, currently recognised as a phenotype of neovascular age-related macular degeneration (n-AMD). However, as PCV tends to develop at a younger age and associated with different clinical features from n-AMD, some controversy exists regarding if PCV should be classified as a separate disease entity [1]. PCV has been reported to be more prevalent in East Asian populations with reported rates of 22.3–61.6% among patients with suspected n-AMD [2], whilst the proportion in Caucasian individuals was estimated to be 8.7% [3]. However, the prevalence of PCV in Caucasian is probably underestimated, especially as indocyanine-green angiography (ICG-A) is not performed routinely [4].

The treatment of choice for PCV is contentious. Photodynamic therapy with verteporfin (PDT) has been widely reported as a successful treatment in inducing polypoidal lesion regression [5, 6]. Intravitreal injection of anti-vascular endothelial growth factor (VEGF), including ranibizumab and aflibercept, has been shown to deliver an optimal visual outcome in patients with n-AMD, including PCV [7]. The LAPTOP study revealed the superiority of intravitreal injection of ranibizumab monotherapy in visual outcome over PDT monotherapy at 24-months [8], whilst PDT was more effective in regressing polypoidal lesions in the EVEREST trial [5]. Combining the two treatment options could theoretically deliver greater functional and anatomical improvements by compounding the benefit of polyp closure induced by PDT and maintenance of vision brought by anti-VEGF. Although some evidence suggested that anti-VEGF monotherapy was non-inferior to combination therapy [9–11], the recent EVEREST-II study established that PDT with adjunct ranibizumab provided favourable outcomes in visual gains and polyp regression with less injection required, compared to monotherapy [12].

Whilst most evidence on the efficacy of combination therapy for PCV was derived from studies in the Asian population with treatment-naive eyes, there is limited data regarding the use of PDT as a rescue in eyes refractory to anti-VEGF monotherapy [13, 14]. The current study aims to describe the clinical outcomes of a British cohort of patients with PCV, treated with anti-VEGF injection plus either early PDT administered at the beginning, or deferred PDT as a rescue.

## Methods

### Study design and population

We conducted a retrospective chart review of patients with PCV that received verteporfin PDT at Macular Treatment Centre (MTC), Manchester Royal Eye Hospital, between January 2016 to December 2019. MTC is a tertiary referral centre for the northwest of England. Patient data were extracted from electronic medical records (Medisoft, Leeds, the UK) and all patient identifiable information were anonymised. Only patients that had at least 18 months of follow-up were included. Eyes with pre-existing visually-impairing pathology such as glaucoma, retinal vessel occlusion, diabetic retinopathy, uveitis, rhegmatogenous retinal detachment or cataracts were excluded. Each eye was analysed individually, irrespective of the status and treatment received in the other eye. The study was approved by the clinical audit department of the Manchester Royal Eye Hospital and conformed to the standards described in the Declaration of Helsinki.

### Clinical examination and investigations

All patients underwent the following ophthalmic examinations at first presentation and during follow-up visits: best-corrected visual acuity (BCVA) measured in early treatment diabetic retinopathy study (ETDRS) letters, slit-lamp biomicroscopy of the fundus, optical coherence tomography (OCT) imaging (Spectralis, Heidelberg Engineering, Heidelberg, Germany; or Topcon 3D OCT-2000, Topcon Corporation, Tokyo, Japan). OCT-Angiography was performed in all patients prior to starting treatments. ICG-A was performed in all patients either at the outset if there was clinical suspicion of a polypoidal lesion, or after a period of sub-optimal visual or anatomical response to frequent anti-VEGF injections. Diagnosis of PCV was established based on the presence of angiographic features described by the EVEREST study group [15].

### Treatment strategies

In the presence of subretinal (SRF) or intraretinal fluid (IRF) or sub-macular haemorrhage, intravitreal anti-VEGF monotherapy typically commenced as first-line treatment. Anti-VEGF agents used included ranibizumab 0.5 mg/0.05 mL or aflibercept two mg/0.05 mL. Treatment regimens comprised of fixed-dosing, treat-and-extend, and pro re nata (PRN). Once PCV was diagnosed, patients were offered PDT at the outset or as ‘rescue or add-on’ treatment. PDT was performed with verteporfin (Visudyne, Novartis, Basel, Switzerland) according to the protocol of the EVEREST II study [12].

Subjects included in this study were stratified based on the timing of PDT, i.e., patients that received PDT at the outset plus intravitreal anti-VEGF injections combination therapy (Initial-PDT group), and patients that received PDT as “rescue or add-on” after suboptimal response to first-line anti-VEGF monotherapy (Deferred group). All patients were treatment-naïve prior to receiving the treatments described and analysed below. Clinical examinations and OCT were performed at each visit. The indication for injections following PDT were: (1) help to stabilise vision and increase treatment interval in eyes that had achieved polyp closure; (2) prevent sub-macular haemorrhages; (3) control disease activity when worsening exudative changes e.g., SRF/IRF were noted. Further PDT were indicated if residual or new polyps were found. Ultimately, decision on retreatment at each visit, follow-up intervals and treatment modality depended on the managing retinal specialist’s discretion and patient’s preference.

### Outcome measures

The primary outcome measures were the changes in anti-VEGF treatment burden and BCVA before and after PDT. Other outcome measures included the proportion of eyes achieving an injection interval ≧12 weeks (including PRN and no more injection required), the proportion of eyes gaining ≧ 5, 10 or 15 letters, and attaining BCVA ≥ 70 letters. Moreover, we performed a multivariate logistic regression analysis to identify the baseline parameters for predicting good visual outcome (BCVA ≥ 70 letters) at 18 months and anti-VEGF injection interval ≥12 weeks apart at the final visit. Potential explanatory variables including age, time to correct identification of polypoidal lesions (diagnostic delay), location of polyps, anti-VEGF agent, BCVA at first presentation, baseline BCVA before PDT, and the number of anti-VEGF injections before PDT were first calculated by a univariate logistic model. The parameters with potential association (*p* < 0.2) were kept in the final multivariate analysis.

### Statistical analysis

Statistical analysis was performed using SPSS (Chicago, IL, USA). The normality of data was assessed using histograms and Kolmogorov–Smirnov/Shapiro–Wilk tests. Paired or two-tailed Student’s *t* test were used to analyse parametric continuous variables, Wilcoxon signed-rank test and Mann–Whitney test for non-parametric continuous variables, and Fisher’s exact test for categorical data. The Levene’s test was used to confirm homogeneity of variances is met. *P* values < 0.05 were classified as significant.

## Results

### Demographics

Baseline characteristics of the included patients are shown in (Table 1). A total of 61 PCV cases treated with combined therapy of PDT and anti-VEGF intravitreal injection met the study inclusion criteria. After excluding 5 cases due to pre-existing visually impairing pathology and 2 cases with less than 18-months follow-up, 53 eyes from 46 patients were included in this analysis. The most common presenting diagnosis before PCV being identified was n-AMD (*n* = 36, 81.8%) and central serous chorioretinopathy (*n* = 2, 4.5%). The mean (±SD) time to recognition of polypoidal lesion was 26.6 (21.7) months in these eyes. PCV was diagnosed at initial presentation in 15 eyes (34%), nine of which received PDT in combination with anti-VEGF as first-line therapy (Initial-PDT group).

**Table 1:** Baseline characteristics of the eyes included in the analysis.

	Initial-PDT		Deferred	
Eyes, *n*	9		44	
Age (Mean ± SD)	74.8 (10.1)		76.4 (7.1)	
Sex (Male)	2.0	22.2%	11.0	25.0%
Ethnicity				
Caucasian	7	77.8%	37	88.1%
Afro-Caribbean	0		3	7.1%
East Asian	0		1	2.4%
South Asian	2	22.2%	1	2.4%
Laterality (Right eye)	5.0	55.6%	19.0	43.2%
Initial Diagnosis				
CSCR	0.0		2.0	
Neovascular AMD	0.0		36.0	
PCV	9.0		6.0	
Time to PDT (Months, Mean±SD)	0.0		24.2 (21.4)	
OCT features at baseline				
SRF	8	88.9%	43	97.8%
IRF	1	11.1%	13	29.5%
PED	7	77.8%	44	100%
Anti-VEGF:				
Ranibizumab	1.0	11.1%	7.0	15.9%
Aflibercept	8.0	88.9%	18.0	40.9%
Switch from Ranibizumab to Aflibercept	0.0		19.0	43.2%
Lesion site:				
Peripaillary	3.0	33.3%	9.0	20.5%
Juxtafoveal	4.0	44.4%	30.0	68.2%
Extrafoveal	2.0	22.2%	5.0	11.4%
BCVA at presentation (Mean ± SD)	73.8 (17.2)		63.6 (12.3)	
Baseline BCVA before PDT (Mean ± SD)	73.8 (17.2)		58.7 (13.3)	
Total number of injections before PDT (Mean ± SD)	0.0		16.8 (11.4)	

*SD* standard deviation, *CSCR* central serous chorioretinopathy, *AMD* age-related macular degeneration, *PCV* polypoidal choroidal vasculopathy, *PDT* photodynamic therapy, *Anti-VEGF* anti-vascular endothelial growth factor, *BCVA* best corrected visual acuity.

Overall, forty-four eyes were included in the Deferred group, while nine were in the Initial-PDT group. Baseline demographic data were similar between the two groups. The Deferred group presented with poorer BCVA (mean letters 63.6 ± 12.3 vs 73.8 ± 17.2, *p* = 0.04) and had worse baseline BCVA at the time of PDT (58.7 ± 13.3 vs 73.8 ± 17.2, *p* = 0.006). Intravitreal aflibercept was used in a similar proportion of patients between the two groups (88.9% Initial-PDT group and 40.9% of Deferred group). The distribution of locations of polyps was similar between the two groups. The mean follow-up time after PDT was 28.3 (16.7) months, and 53 (100%), 49 (92.5%) and 37 (69.8%) cases completed an 18-, 24- and 36-months follow-up respectively.

### Anti-VEGF treatment burden before and after PDT

**Fig. 1:**
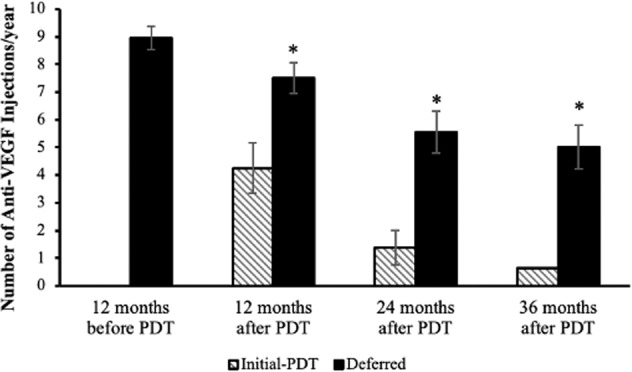
The number of anti-VEGF injections required per year (mean ± standard error of the mean) 12-months before PDT (Deferred group), and at 12-months, 24-months and 36-months after PDT (Initial-PDT and Deferred groups). Only patients completed 36-month follow-up were presented. In the Deferred group, the mean number of anti-VEGF injections required over 12-, 24-, 36-months after PDT were all significantly less than that over the 12-months before PDT (*p = 0.002, 0.001, 0.001 respectively).

The median time interval from initial presentation to the day of PDT in the Deferred group was 22.0 months (Interquartile range, 7.0–33.5). During this time, these patients received a mean of 9.4 (2.3) injections per year. At time of PDT, 35 eyes (79.6%) were on a fixed-dose 4-weekly intravitreal anti-VEGF treatment regimen, 6 eyes (13.6%) were on 6-weekly regimen, 1 eye (2.3%) each was on 8-weekly and 12-weekly regimen. Overall, 33 eyes (62.3%) were on treat-and-extend anti-VEGF regimen at the final follow-up visit after PDT, 13 eyes (24.5%) were on PRN, and 7 eyes (13.2%) were on fixed-dosing. The mean number of injections per year after PDT was significantly reduced to 7.2(3.6) (*p* < 0.001), in other words, the mean time interval between each anti-VEGF injections was extended from 1.4 (0.4) to 2.1 (1.5) months (*p* < 0.001). The mean number of anti-VEGF injections required over 12-, 24-, 36-months after PDT were all significantly less than that over the 12-months before PDT (*p* = 0.002, 0.001, 0.001 respectively) and showed a downward trend (Fig. 1). However, eyes in the Initial-PDT group required significantly fewer anti-VEGF injections per year after receiving PDT compared to the Deferred group (3.3 ± 2.9 vs 7.2 ± 3.6, *p* = 0.004). Moreover, a significantly higher proportion of eyes in the Initial-PDT group achieved an injection interval of 12 weeks or more at the final visit compared to the Deferred Group (88.9% vs 40.9%, *p* = 0.011). Overall, 3 eyes required one additional session of PDT, and 1 eye required two additional sessions. They were all in the Deferred Group.

### Visual outcome after combination therapy with PDT and anti-VEGF

Before receiving “rescue” PDT, patients in the Deferred group received monotherapy of intravitreal anti-VEGF injection as the first-line treatment. Mean BCVA in this group dropped from 63.6 (12.3) at first presentation to 58.7 (13.3) on the day of PDT (*p* = 0.019), despite regular anti-VEGF injections. After PDT, eyes in the Deferred group experience improvement in BCVA from 58.7 at baseline to 63.3 (15.0) at 6 months, 66.0 (16.9) at 12 months, and 63.8 (16.9) at 18 months follow-up (*p* = 0.001, 0.004, and 0.001, respectively), but no significant increase was observed at these time points in the Initial-PDT group (73.8 ± 17.3 letters at baseline, 74.9 ± 17.3, 74.8 ± 16.5 and 76.3 ± 17.9 at 6-, 12- and 18-months, respectively [*p* = 0.812, 0.859, 0.310]). There was no significant difference in BCVA at 18-months follow-up post-PDT between the Initial-PDT and Deferred groups (*p* = 0.163).

**Fig. 2:**
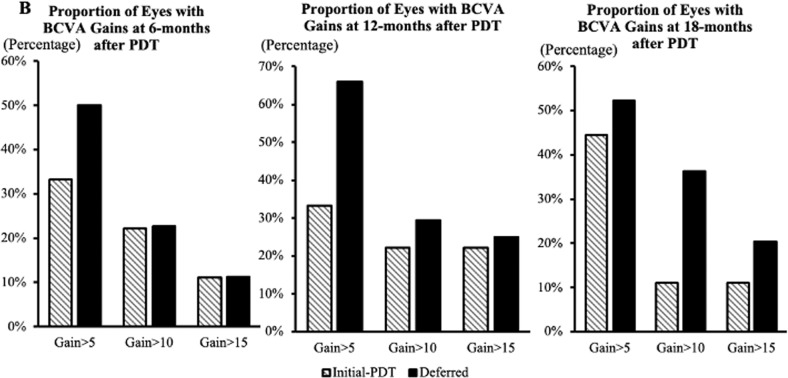
Proportion of BCVA gain (letters, by category) at specific time-points after PDT (Initial-PDT and Deferred group). All eyes were included. A higher proportion of eyes gained ≥ 5, 10 and 15 letters in the Deferred group than the Initial-PDT group at 6-, 12-, and 18-months after PDT.

At 18-months follow-up after PDT, the mean VA change from baseline BCVA was similar in both groups, with eyes in Deferred and Initial-PDT group gaining 5.1 (13.5) and 2.9 (8.7) letters, respectively (*p* = 0.639). Moreover, a higher proportion of eyes gained ≥5, 10 and 15 letters in the Deferred group than the Initial-PDT group (Fig. 2). In the Deferred group, 20 eyes (45.5%) achieved good vision with BCVA ≥ 70 letters at 18-months follow-up, which was significantly more than the baseline (12 eyes, 27.3%; *p* = 0.039). A drop in BCVA of ≥ 5 letters was observed in 3 eyes (33.3%) of the Initial-PDT group and 7 eyes (15.9%) of the Deferred group at 18-month follow-up (*p* = 0.346). At 18-months follow-up, “Dry macular” (no SRF/IRF) was achieved in 8 (88.9%) and 20 (36.4%) eyes from the Initial-PDT and Deferred groups, respectively; PED height was reduced in all 9 eyes of the Initial-PDT group, and 25 (56.8%) eyes of the Deferred group.

**Fig. 3:**
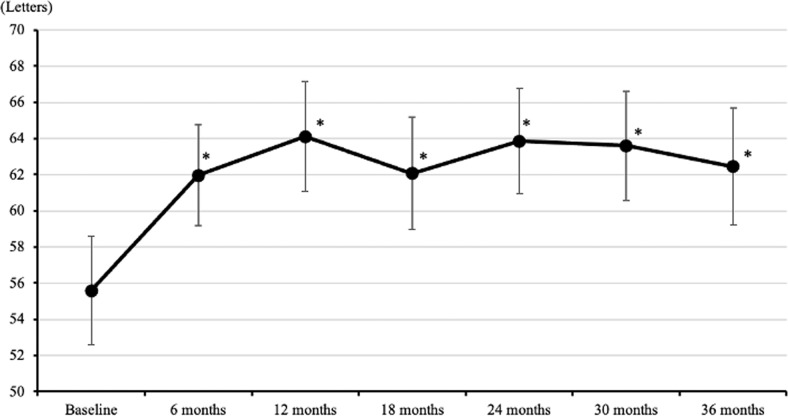
Mean BCVA in ETDRS letters (mean ± standard error of the mean) up to 36-Month after PDT (All the patients who had completed 36 months follow-up). Baseline is defined as the last BCVA recorded prior to PDT. *The *p* values between the baseline and each time point were all <0.05.

In the 37 eyes that completed a longer-term 36 months follow-up, mean BCVA peaked at 12 months after PDT, but there were significant improvements compared to baseline at all time-points measured (*p* < 0.05 at all time points) (Fig. 3). At 36 months after PDT, 12 eyes (32.4%) showed an improvement in BCVA of ≥10 letters, and 14 eyes (37.8%) retained good BCVA ≥ 70 letters, while 7 eyes (18.9%) showed a deterioration in BCVA ≥ 5 letters.

### Predictors

We performed univariate and multivariate analysis to investigate the parameters predictive of visual outcome and injection interval. Baseline BCVA before PDT was positively associated with achieving BCVA ≥ 70 letters; each letter increases in baseline BCVA corresponded to the eye being 112% more likely (OR = 1.12) to achieve BCVA ≥ 70 at 18 months follow-up (*p* = 0.006). The number of anti-VEGF injections/year before PDT negatively influence the likelihood of achieving injection interval ≥12 weeks apart, i.e., for every additional injection per year before PDT, the re-treatment interval between each injection was 72.4% less likely (OR = 0.724) to achieve 12 weeks or longer apart after PDT (*p* = 0.006) (Table 2). Age, gender, BCVA at presentation, diagnostic delay, choice of anti-VEGF agents and polyp locations were not significantly associated with BCVA ≥ 70 letters at 18 months follow-up, nor anti-VEGF injection treatment interval ≥12 weeks apart at the final visit.

**Table 2:** Result of multivariate logistic regression analyses of factors predictive for good visual outcome (BCVA ≥ 70 ETDRS letters) at 12-months after PDT, and injection-interval of ≥12 months apart.

Factors	Extension of Injection Interval to ≥12 weekly		
	OR	95% CI	*P* value (multivariate)
BCVA at baseline before PDT	1	0.95–1.05	0.988
Total *n*. of injection before PDT	1.00	0.93–1.07	0.977
Injection/year before PDT	0.72	0.58–0.91	0.006
Polyp location			
Peripapillary	3.51	0.22–57.18	0.378
Juxtafoveal	1.48	0.14–15.76	0.745
Anti-VEGF agents			
Eylea	0.25	0.05–1.38	0.112
Lucentis	0.16	0.01–2.95	0.217

Factors	Good Visual Outcome of BCVA ≥ 70 Letters		
	OR	95% CI	*P* value (multivariate)
Age	0.95	0.84–1.07	0.370
BCVA at presentation	1.01	0.93–1.10	0.769
BCVA at baseline before PDT	1.12	1.03–1.21	0.006
Total n. of injection before PDT	0.98	0.91–1.06	0.588
Injection/year before PDT	1.08	0.85–1.38	0.531
Polyp location			
Peripapillary	1.86	0.15–22.78	0.628
Juxtafoveal	2.22	0.20–24.37	0.514
Anti-VEGF agents			
Lucentis	1.37	0.14–13.22	0.788
Combination	1.47	0.22–10.12	0.693

*OR* odd ratio, *CI* confidence interval, *BCVA* best corrected visual acuity, *PDT* photo dynamic therapy, *VEGF* vascular endothelial growth factor.

## Discussion

To our knowledge, this is the first study that describes the efficacy of PDT in treating patients with PCV already receiving regular anti-VEGF injections in the UK, as most existing studies focus on assessing the efficacy of PDT in treatment-naïve patients. Our real-world experience demonstrated that PDT effectively improved visual outcome and reduced the anti-VEGF treatment burden, even in patients who had experienced a period of suboptimal disease control by anti-VEGF monotherapy.

Multiple pivotal clinical trials had established the efficacy of combination therapy with intravitreal anti-VEGF injections and PDT for PCV. In the PLANET study, participants treated with aflibercept followed by rescue PDT at 12th weeks showed a BCVA gain of 9.1 letters at 24-month [9], while the mean BCVA gains at 12- and 24-month after combination therapy of PDT with ranibizumab were shown to be 8.1(ref. [16]) and 9.6 (ref. [12]), respectively. Overall, the mean BCVA gains in our study were 4.7 letters at 18-month after combination therapy, which was fewer than those reported by more extensive trials. Moreover, a smaller proportion of our patients achieved BCVA gain ≥10 and ≥15 letters (32.1% and 18.9%, respectively) compared to current literature [12]. Whilst most clinical trials enroled patients of Asian descent who were treatment-naïve, and PDT was performed at the outset, the majority of our cohort received anti-VEGF monotherapy as the first-line treatment; rescue PDT was only offered in eyes refractory to frequent injection regimen. The median time-interval from starting the first injection to the day of PDT in our patients was 22.0 months, therefore our treatment regimen inherently differed from the “early/initial” PDT in EVEREST-II [12], or even the “late PDT” in PLANET and Fujisan Study [9, 16]. Ratanasukon et al. postulated that multiple anti-VEGF injections could change the histological structures of the polypoidal lesion, modulating its response to PDT, and ultimately dampened the treatment benefit [17]. Besides, some of the patients selected for PDT in our study might have suffered from irreversible anatomical damage due to long-standing sub-optimal control of disease activity by anti-VEGF monotherapy. Additional visual benefit from adding PDT in these eyes would have been limited. Nonetheless, although not to the same extent as reported by published trials, our patients experienced significant BCVA gains after the combination therapy.

Interestingly, although the eyes treated with “initial” PDT had better visual outcome than those with deferred PDT at 18-month follow-up, the Deferred group achieved more considerable numerical gain in letters. As the baseline BCVA for the Initial-PDT group (73.8) was significantly higher than the Deferred group (58.7), the potential for the Initial-PDT group to gain more letters may be kerbed by a ceiling effect. Focusing on the BCVA gains in eyes that have completed the longer-term 36-months follow-up, it is also noteworthy that there was sustained improvement in visual acuity over a 3-years period after PDT, all the while the mean number of anti-VEGF injections required each year continued to fall. This further suggests that the potential benefit of PDT on visual outcome and treatment burden is likely long-lasting.

Although the use of PDT combined with anti-VEGF injection is recommended for PCV by some trials, there are reports that anti-VEGF monotherapy can also achieve good visual outcomes at 12 months [18, 19]. Some comparative analyses have demonstrated the superiority of ranibizumab or aflibercept monotherapy in functional or anatomical advantages over combination therapy [9–11, 20]. However, contrary to these reports, our patients in the Deferred group experienced significant drops in mean BCVA when they were treated with standard anti-VEGF monotherapy only. Unlike clinical trials, real-world studies like this report lack standardised treatment protocols and criteria, explaining the discrepancy. Moreover, our cohort in the Deferred group consisted of patients who received PDT due to inadequate response to anti-VEGF monotherapy; falling visual acuity in these patients is not surprising.

The burden of frequent anti-VEGF injection has a potential impact on both patients and the healthcare system; therefore the number of injections required per year was chosen as one of the outcome measures in this report. We showed that the number of injections required in the Deferred group significantly reduced from 9.4 to 7.2 per year after PDT. This finding concurs with the outcomes reported by smaller case series in the US [13] and Switzerland [14]. Although the participants in the combination therapy group in the EVEREST-II trial required less injection per year (4/year) than our Deferred group, it is worthy of mention that the patients in clinical trials were all treatment-naïve and received PDT at the outset [12]. Similarly, the treatment-naïve patients (Initial-PDT group) in our study received a mean of 3.3 injections/year after PDT, comparable to those reported in the EVEREST-II trial. Furthermore, a higher percentage of eyes in the Initial-PDT group than the Deferred group succeeded in extending injection interval to ≥12 weeks apart. These findings emphasised the importance of prompt diagnosis of PCV and early use of combination therapy.

We investigated the factors predictive of an eye achieving BCVA ≥ 70 letters at 18-month follow-up, and the most significant predictor was baseline BCVA before PDT. Our finding concurs with current literature, which suggests that baseline BCVA is an essential clinical factor determining the final visual outcomes at one year after PDT [21, 22]. However, other previously reported predictors, including age, location of polypoidal lesions, and injection frequency, were not found to be significantly associated with visual outcome in our cohort [22, 23].

This study is limited by its retrospective design and single-centre cohort. It is worthy of mention, though, despite being a single-centre study, patients are managed by ten medical retina specialists, the data presented thus provides an accurate representation of real-world outcome in a large tertiary referral centre. All patients underwent OCT and OCT-Angiography at the first visit. However, as ICG-A is the gold standard imaging modality for diagnosing PCV, the lack of obligatory ICG-A at initial consultation inevitably led to diagnostic delay. Moreover, as ICG-A was not mandated during follow-up, polyp regression could not be assessed. Within majority of NHS ophthalmology services, it is not possible to perform baseline ICG-A in every patient who has presented with n-AMD. Although less than ideal, the diagnostic procedures described in this study nevertheless represent real-world practices, especially in centres where ICG-A is not routinely available [13]. Diagnostic criteria for PCV utilising the non-invasive and widely-available OCT may improve polyp recognition in settings in which ICG-A is not performed routinely [24]. Furthermore, the anti-VEGF agents used and the switch in between agents were not examined separately. Hence cautions should be taken when drawing a direct comparison with the outcome of larger trials. Nonetheless, we feel this factor did not meaningfully influence the robustness of the outcome, particularly concerning the reduction of treatment burden after PDT.

In summary, we presented real-world visual outcome and treatment burden for PCV treated with anti-VEGF injections combined with PDT. We confirmed the beneficial role of PDT as a rescue therapy in the long-term management of PCV, particularly in eyes that had experienced a significant period of prior exposure to anti-VEGF monotherapy. It also highlights that in case of strong clinical suspicion of PCV, prompt PDT may help reduce the treatment burden, particularly in countries where there is limited public financial coverage for anti-VEGF treatments. We further confirmed that baseline BCVA was an important factor predictive of good visual outcome. Echoing the findings in EVEREST-II, our experience provides real-world evidence that supports the use of a combination of anti-VEGF with PDT in PCV.

## Summary

### What was known before

- Combination therapy of photodyanmic therapy (PDT) and anti-vascular endothelial growth factor (anti-VEGF) is effective for polypoidal choroidal vasculopathy (PCV) - Most current evidence derived from studies in the Asian population with treatment-naive eyes

### What this study adds

- PDT is effective in improving vision and reducing treatment burden in eyes with PCV refractory to a significant period of anti-VEG monotherapy - Early PDT provides superior outcomes compared to delayed PDT, highlighting the importance of prompt investigation and treatment in case of strong clinical suspicion
